# Nuclear PTEN’s Functions in Suppressing Tumorigenesis: Implications for Rare Cancers

**DOI:** 10.3390/biom13020259

**Published:** 2023-01-30

**Authors:** Casey G. Langdon

**Affiliations:** 1 Department of Pediatrics, Darby Children’s Research Institute, Medical University of South Carolina, Charleston, SC 29425, USA; langdonc@musc.edu; Tel.: +1-(843)-792-9289; 2 Hollings Cancer Center, Medical University of South Carolina, Charleston, SC 29425, USA

**Keywords:** nuclear PTEN, PTEN, PTEN hamartoma tumor syndrome, subcellular localization, nuclear import, post-translational modifications, genetically engineered mouse models, DNA damage, oncogenic transcriptional regulation

## Abstract

*Phosphatase and tensin homolog* (*PTEN*) encodes a tumor-suppressive phosphatase with both lipid and protein phosphatase activity. The tumor-suppressive functions of PTEN are lost through a variety of mechanisms across a wide spectrum of human malignancies, including several rare cancers that affect pediatric and adult populations. Originally discovered and characterized as a negative regulator of the cytoplasmic, pro-oncogenic phosphoinositide-3-kinase (PI3K) pathway, PTEN is also localized to the nucleus where it can exert tumor-suppressive functions in a PI3K pathway-independent manner. Cancers can usurp the tumor-suppressive functions of PTEN to promote oncogenesis by disrupting homeostatic subcellular PTEN localization. The objective of this review is to describe the changes seen in PTEN subcellular localization during tumorigenesis, how PTEN enters the nucleus, and the spectrum of impacts and consequences arising from disrupted PTEN nuclear localization on tumor promotion. This review will highlight the immediate need in understanding not only the cytoplasmic but also the nuclear functions of PTEN to gain more complete insights into how important PTEN is in preventing human cancers.

## 1. Introduction

The ability for cells to grow uncontrollably is a central tenet underscoring what makes a cancer cell different than the normal cell from which it arose. Indeed, normal cells and tissues have evolved many checkpoints to safeguard themselves from undergoing oncogenic transformation. However, in cancer cells, these safeguards have failed. Cancer cells have evolved distinct mechanisms to evade intrinsic and extrinsic tumor-suppressive signals normally present in non-transformed cells. These cancer hallmarks have formed our foundational understanding of tumorigenic biology [1].

One of the most frequently lost tumor suppressors in human cancer is the Phosphatase and tensin homolog (PTEN) [2]. PTEN is required for life and the formation of all three germ layers in the developing embryo; homozygous, germline loss of PTEN leads to embryonic lethality [3,4,5,6]. However, when only one PTEN allele is lost, PTEN acts as a haploinsufficient tumor suppressor; tumors are found within the thyroid, endometrium, liver, prostate, thymus, and gastrointestinal tracts, and lymphomas of germline heterozygous mice [3,4,5]. These early murine germline-mutant tumor models closely mimic the multi-organ involvement and pathophysiology of several, rare human cancer predisposition syndromes-Cowden syndrome, Bannayan–Riley–Ruvalcaba Syndrome, adult Lhermitte-Duclos disease (dysplastic cerebellar gangliocytoma)—collectively known as PTEN hamartoma tumor syndromes (PHTS) [7].

Loss of PTEN activity is achieved through a variety of mechanisms in human cancers including point mutations (several summarized in Table 1), chromosomal deletions, promoter hypermethylation leading to transcriptional repression, and altered protein stability dynamics [8]. Many of these genetic, epigenetic, and post-translational PTEN alterations change the enzymatic activity of PTEN. PTEN expression is also regulated by many different RNA binding proteins, microRNAs (miRNAs), and other non-coding RNAs. Musashi-1 (MSI1) and Musashi-2 (MSI1) are RNA binding proteins that negatively regulate PTEN expression in gliomas and colonic epithelium, respectively [9,10]. Alternative polyadenylation of the *PTEN* 3′UTR (untranslated region) can also increase mRNA stability [11]. PTEN is negatively regulated by many different miRNA, but positively regulated by many competing endogenous (ceRNAs) suggesting a dynamic post-transcriptional regulatory landscape for PTEN expression [12]. Several miRNAs, including miR-18a, miR-21, miR-26a, and miR-92a, negatively regulate PTEN expression across breast cancers, hepatocellular carcinoma, high-grade glioma, and colorectal cancers, respectively [13,14,15,16]. Other non-coding RNAs, including ceRNAs such as the PTEN pseudogene *PTENP1* and long non-coding RNAs (lncRNAs) like *HOTAIR* positively regulate PTEN expression [17,18,19,20,21].

PTEN has five different structural domains-an N-terminal phosphatidylinositol-4,5-bisphosphate (PIP2)-binding domain (PBD), a phosphatase catalytic domain, a C2 domain, and a C-tail domain that leads into a PSD-95/Dlg/ZO1 (PDZ)-binding domain (PDZ-BD) [53]. The PBD domain is important for membrane binding and PTEN catalytic activity by binding PIP2 leading to allosteric PTEN activation [54,55,56]. PTEN is a phosphatase and dephosphorylates both lipid and protein substrates [33,57,58,59]. PTEN can dephosphorylate serine, threonine, and tyrosine residues [33,59]. As a lipid phosphatase, PTEN principally catalyzes the formation of PIP2 from the substrate phosphatidylinosital-3,4,5-triphosphate (PI(3,4,5)P_3_) [57]. The ability to be both a lipid and protein phosphatase is most likely due to an enlarged activation site in the phosphatase domain that can bind the larger PI(3,4,5)P_3_ residue [60]. Mutations within the phosphatase domain are critical for destroying PTEN enzymatic functions. C124S mutations are catalytically inactive [33]. The lipid and protein phosphatase functions for PTEN can be demarcated by G129E (lipid phosphatase deficient) or Y138L (protein phosphatase deficient) [33,36]. The N-terminal phosphatase domain and the C2 domain also form a minimal enzymatic unit that is responsible for facilitating the formation of PIP2 [61]. The C2 domain binds phospholipids and has high affinity for cellular membranes [60]. The C-tail domain has several residues whose phosphorylation controls PTEN activity. This includes the formation of a phosphorylated “belt” around the phosphatase and C2 domains that keeps PTEN in a “closed” confirmation preventing the hydrolysis of PI(3,4,5)P_3_ and leading to membrane dislodgement [62,63]. This closed confirmation prevents the PDZ-BD from interacting with PDZ containing proteins [63]. Disrupting these PDZ-BD and PDZ interactions may lead to dysregulated subcellular localization as seen with MAST2 (microtubule associated serine/threonine kinase 2), PTEN, and the rabies viral glycoprotein or with MAGI-1b, -2, or -3 (membrane-associated guanylate kinase) scaffolding proteins and PTEN [54,64]. When mapping known germline mutations to the structural domains found within PTEN, most of the amino acid substitutions are found within PTEN’s phosphatase domain, but there are still many mutations observed throughout the entire protein length [60,65]. This suggests both phosphatase-dependent and -independent functions for PTEN in tumor suppression.

Inside the cell, PTEN is found within the cytoplasm, the nucleus, the plasma membrane, mitochondria, and endoplasmic reticulum where its interaction partners are distinct. Understanding how disrupted PTEN subcellular localization contributes to tumorigenesis is incomplete, and more work is needed to determine the full impact of PTEN loss in oncogenesis [2,66,67,68,69,70,71,72,73,74,75,76]. This review will focus on nuclear and cytoplasmic PTEN localization. Furthermore, this review will show how PTEN localization in the cytoplasm and the nucleus is regulated, how PTEN is translocated into the nucleus, and what PTEN’s nuclear tumor-suppressive functions are. A special focus of the review will be to show how studying nuclear PTEN’s biology in rarer cancers can provide significant advances in examining the full complement of PTEN’s anti-neoplastic functions. By taking a more holistic approach to discern these functions in rare cancers, a major aim of the review is to show the potential synergistic effect between rare cancer research and nuclear PTEN function discovery [77].

## 2. PTEN Expression and Activity Loss in Rare Cancers

PHTS is a rare collection of syndromes defined by the loss of PTEN function through germline mutations in PTEN or in mutations of other genes leading to PTEN activity changes [7,78,79,80]. Aside from cancer predisposition, these patients present frequently with macrocephaly, multinodular goiter, developmental delay and are frequently diagnosed along the autism spectrum [81,82,83]. The prevalence for PHTS is 1 in 200,000 people, but this may be underestimated given the phenotypic variation seen in PHTS patients [82,84]. Many different cancers are observed in patients with PHTS, in both pediatric and adult populations, including breast, endometrial, thyroid, colorectal, and renal cancers plus melanoma [85]. This phenotypic variation is recently described in an index cohort of European PHTS patients with pathogenic or likely pathogenic PTEN alterations, where early-onset PHTS correlated with missense variants and later-onset PHTS correlated with truncating mutations [82]. This corresponds with an increased risk for developing breast cancer, a later onset PHTS-related cancer, when patients had a truncating mutation compared to a missense variant [85]. 

Two rare brain tumors, meningiomas (8.25%) and gangliocytomas (1.8–15%), also have high incidence rates among PHTS patients; adult-onset dysplastic cerebellar gangliocytomas, or Lhermitte-Duclos disease, is specifically enriched for PTEN mutations (83%) [7,86,87]. In the brain, PHTS-related PTEN mutations such as R234Q can form tumors across multiple lineages, such as gliomas, within the same patient [40,88].

Several missense variants seen in PHTS are also sufficient to cause mislocalization of PTEN out of the nucleus. The PTEN K289E mutation, seen in some Cowden syndrome patients, causes PTEN to be restricted to the cytoplasm [22]. Originally found in glioblastomas, PTEN K13E was the first lysine 13 mutation discovered and, like K289E, was also implicated in subcellular localization changes-shifts in PTEN protein away from the plasma membrane and out of the nucleus although this mutation is not seen in PHTS patients [22,55,89]. Other PTEN missense variants-F241S, D252G, W274L, and N276S-associated with autism spectrum disorders (ASD) also cause nuclear exclusion in neurons; all four of these mutations are cataloged in COSMIC (Catalog of Somatic Mutations in Cancer) [44]. Three of these mutants-F241S, D252G, and N276S-also are far less stable, although catalytically functional [30]. Other PHTS-associated mutations, such as T26I, P95R, Y177N, Q261E, T277A, and D310G, are also nuclear excluded [25]. In contrast, when examining the localization of other PTEN mutations found in autistic PHTS patients, Wong et al. found that G44D, H123Q, E157G, and D326N are more nuclear localized than wild-type PTEN with no relationship between localization and stability or phosphatase activity [26]. Many of these PTEN germline mutations found in PHTS patients also affect protein stability [26,30,48]. For example, I101T has significantly less PTEN protein expression than wild-type PTEN, lipid phosphatase activity, and reduced nuclear:cytoplasmic (N:C) PTEN protein ration [30,31]. The C124S PTEN inactivating mutation may also have decreased protein stability compared to wild-type or lipid phosphatase-dead G129E, although there are disparate claims about the stability of the C124S mutation [34,35]. Indeed, PHTS-associated mutation G129E and endometrial cancer-associated C124S mutations can form heterodimers with wild-type PTEN and exert a dominant-negative function against wild-type PTEN [35]. These delineations in determining the tumor-suppressive mechanisms lost when PTEN is mutated are key due to the wide mutational plethora observed and many ways that PTEN function can be disrupted. As new PTEN PHTS mutations are discovered, empirical determinations of enzymatic activity, localization, and stability profiles must be completed for the field to fully comprehend the complete spectrum of PTEN functions that are undermined in PHTS. Therefore, understanding the functions of rare PTEN mutant alleles found in cancers and developmental disorders will allow for a more personalized approach to patients who have PHTS or somatic PTEN mutations.

Given PTEN has so many reported variants, large-scale analyses of variant function are needed to fully encapsulate the mechanisms underlying PTEN tumor suppressor loss. Multiplexed assays such as VAMP-seq (Variance Abundance by Massively Parallel sequencing) can help delineate if a variant acts in a dominant negative fashion or is in low abundance [90]. Furthermore, to empirically determine how PTEN mutations alter protein function, Post and colleagues cataloged the effects of these mutations through multi-organismal screening assays in yeast, *Drosophila, Caenorhabditis elegans,* and human HEK293T cells on PTEN phosphatase activity, protein stability, and phenotypic behavioral assays [28]. The 106 PTEN variants tested were collected from a mixture of PHTS, somatic cancer, and ASD mutations within the PTEN coding sequence; of those 106, sixty were annotated as pathogenic or likely pathogenic [28]. Together, these methodologies illustrate the need for large-scale pipelines to examine a wide assortment of somatic or germline variants to assess their pathogenicity. This is especially true given the sheer number of PTEN mutations seen in human cancer [91].

Aside from PTHS being a rare, germline, cancer predisposition syndrome, altered PTEN function via missense or truncating mutations, promoter hypermethylation, and chromosomal loss is seen in many cancers, including rare tumors [92]. The US Orphan Drug Act defines a rare disease as one that affects less than 200,000 people in the US (or approximately 1:1500 people), and the EU defines rare diseases as ones that affect 1:2000 people (rarediseases.info.nih.gov). Rare tumors account for up to 25% of all tumors [93,94]. PTEN alterations are commonly found in rare cancers such as glioblastoma, renal cancers (both clear cell and non-clear cell), acral and mucosal melanomas, metaplastic breast cancer, vaginal squamous cell carcinoma, malignant mixed Mullerian tumor, and prostate neuroendocrine carcinoma [92,93,95]. In pediatric cancers, all of which are defined as rare cancers, PTEN activity loss is seen in T-lineage or B-lineage acute lymphoblastic leukemia (T-ALL or B-ALL), osteosarcoma, high grade glioma, medulloblastoma, pilocytic astrocytoma, adrenocortical carcinoma, rhabdomyosarcoma, and pediatric thyroid carcinomas [96,97,98,99,100]. Next-generation DNA sequencing, however, cannot delineate the subcellular location where PTEN protein is found. Instead, understanding the biochemical processes relevant to how PTEN protein gets into the nucleus is critical for determining the consequences of dysregulated PTEN subcellular localization. 

## 3. PTEN Subcellular Localization

### 3.1. Histological Characterization of Nuclear PTEN-from Bench to Bedside

Homozygous PTEN loss in the mouse is embryonic lethal indicating PTEN is essential for development [3,4,5,6]. In the mouse embryo, PTEN expression peaks at embryonic day 11 with continued high expression levels until embryonic day 16 [5]. PTEN is highly expressed in dorsal portions of the mantle layer of the spinal cord, heart, and epidermis [5]. Nuclear PTEN is still seen in adult normal tissue, as observed in non-tumorigenic neurons and vasculature in brains of human glioblastoma multiforme patients [101]. As cells differentiate, PTEN subcellular localization frequently shifts from nucleus to cytoplasm. During neural differentiation, PTEN becomes more nuclear predominant [102]. Brain derived neurotrophic factor (BDNF)-induced differentiation causes an increase in nuclear PTEN levels in differentiated mouse central nervous system neurons [102]. Indeed, nuclear PTEN loss in murine neural stem cells causes stunted neural maturation [103]. In vascular smooth muscle cells (vSMC), nuclear PTEN positively regulates the ability of serum response factor (SRF) to maintain smooth muscle contractility and differentiation; disrupting this interaction leads to vSMC phenotypic switching and more atherosclerotic blood vessels [104]. Nuclear PTEN is also critical in regulating cell cycle entry of vSCMs, with peak nuclear PTEN levels occurring during S phase [105]. This data suggests that PTEN found in nuclei is critical for maintaining and regulating differentiation processes.

Furthermore, as normal cells transform into neoplasia, PTEN subcellular localization frequently shifts back from nucleus to cytoplasm. Nuclear PTEN is found in human normal follicular thyroid cells and myoepithelial breast tissue [106,107]. As these thyroid cells transform into follicular thyroid carcinomas, PTEN nuclear localization is lost in these tumor cells with only minor changes observed in cytoplasmic PTEN expression between normal thyroid tissue and the neoplastic thyroid tissue [106]. Pancreatic islet cells also have strong nuclear PTEN positivity that is lost in endocrine pancreatic tumors [108]. Endometrial and colonic epithelia also have higher nuclear PTEN positivity compared to their respective carcinoma counterparts [71,109,110]. Keratinocytes exhibit higher nuclear PTEN expression compared to basal cell and squamous cell carcinomas [111]. Conjuctival nevi also have significantly higher expression of nuclear PTEN compared to conjuctival melanomas [112]. Therefore, PTEN subcellular alterations seen between normal and transformed tissue highlight the importance of nuclear PTEN in tumor suppression and tumor initiation.

Alterations in PTEN localization during cancer progression are not limited to changes from normal to tumor states, but also with increasing malignancy. Nuclear PTEN is lost in breast cancer brain metastases compared to primary breast tumors [113]. As colonic tumorigenesis progresses from normal colon epithelia to adenoma to aggressive adenocarcinoma through to metastases, nuclear PTEN levels continue to decrease [114]. Angiosarcomas, a rare blood vessel malignancy, also have lower global PTEN expression than benign hemangiomas, but in the cells that remain PTEN expression-positive, the PTEN seen is cytoplasmic [115]. Lung cancers, including adenocarinoma, squamous cell carcinoma, and small cell cancers, all have lower N:C PTEN ratios compared to normal or cancer-adjacent lung tissue; this also includes smaller N:C PTEN ratios in higher grade lung cancers compared to low-grade lung cancer [116]. In both murine and human melanomas, there is a shift to PTEN cytoplasmic localization with increased disease severity [117,118,119]. 

Nuclear and cytoplasmic PTEN partitioning may also be clinically impactful in determining correlates of survival. In lung neuroendocrine tumors, deficits in both nuclear and cytoplasmic PTEN correlated with worse survival, but in all three subtypes tested-small cell carcinoma, typical and atypical carcinoids, and large cell neuroendocrine tumors-nuclear PTEN expression is less robust than cytoplasmic PTEN expression [120]. Breast cancer patients with deficits in nuclear PTEN also had decreased breast cancer related survival than those with intact PTEN localization to the nucleus; indeed, low nuclear/high cytoplasmic PTEN immunohistochemical positivity had the worst survival of all four possible PTEN localization possibilities [121]. Furthermore, human papillomavirus-positive tonsillar cancer cells with intact nuclear PTEN had better overall survival than those patients without nuclear PTEN [122]. Colon cancer patients with PTEN found in the nucleus also fare better than those without any nuclear PTEN [110]. Similar results are also seen in diffuse large B-cell lymphoma and esophageal squamous cell carcinoma-cancer cells with PTEN expressed in the nucleus had better survival [123,124]. More work is needed to determine if these correlative findings can be causally linked to nuclear PTEN localization. These impacts on patient survival dictated by disrupting PTEN subcellular localization homeostasis hints at a robust regulatory system ensuring PTEN can get into the nucleus to exert its tumor-suppressive effects. But how does PTEN enter the nucleus?

### 3.2. Mechanisms Underlying PTEN Nuclear Import

One of the central problems a eukaryotic cell needs to overcome is the ability to systematically, efficiently, and selectively transport proteins produced in the cytoplasm into the nucleus for the correct subcellular functional context [125]. Several mechanisms have been postulated for PTEN nuclear importation. PTEN localization in the nucleus could be mediated by diffusion across the nuclear pore complex [126]. However, the amino acid residues needed for this diffusion also form part of a putative nuclear localization sequence (NLS) [127]. A NLS is a conserved amino acid sequence that serves as a “postal code” for nuclear cargo delivery [125]. NLS are recognized by nuclear transporters and contact nucleoporins to facilitate the delivery of the NLS-containing cargo protein through the nuclear pore complex into the nucleus [125,128]. The first NLS, a seven amino acid peptide Pro-Lys-Lys-Lys-Arg-Lys-Val (PKKKRKV), was discovered through mutagenic analysis of simian virus 40 (SV40) [125,129]. Classical NLS can be characterized as monopartite, such as the SV40 NLS, or bipartite, with two distinct NLS pieces separated by several amino acid residues between the two NLS pieces [125].

Bipartite NLS are not typically contiguous like the SV40 NLS, but instead are found with two distinct clusters of 2–3 positively charged amino acids, separated by a linker region, typically 10–12 amino acids [125,130]. In PTEN, both monopartite and a bipartite NLS have been described. The monopartite NLS for PTEN is found on the N-terminus where there are several positively charged amino acids [127]. This NLS-like sequence is not a classical NLS as seen with SV40 but does bind to Importin-β (IPO1) [127]. IPO1 requires the small GTPase activity of RAN to transfer PTEN from the cytoplasm to the nucleus in glioblastoma cells [127]. However, IPO1 is one of three importins that facilitates nuclear PTEN transport. The other two importins are Transportin-2 (TNPO2 or IPO3) and Importin-11 (IPO11). TNPO2 facilitates the nuclear import of PTEN in hepatoblasts [131]. IPO11 mediates nuclear PTEN import in both murine and human prostate cancer models and mouse lung cancers [132]. IPO11 selectively shuttles ubiquitin-tagged cargos for nuclear import [133]. In PTEN’s case, IPO11 protects PTEN from ubiquitin-mediated degradation [132]. 

As for the bipartite NLS, the NLS must contain amino acids 265–269 with either residues 160–164 or 233–237 [38]. PTEN engages with the Major Vault Protein (MVP) through these NLS in PTEN’s C2 domain, ultimately leading to PTEN shuttling into the nucleus in breast cancer cells [38,134,135]. MVP is a constituent protein of the vault complex, a eukaryotic ribonucleoprotein nanoparticle found mostly in the cytoplasm with only approximately 5% of vaults found in the nuclear membrane near nuclear pore complexes [134,136,137]. MVP overexpression leads to nuclear PTEN accumulation [138]. Nuclear PTEN shuttling can be inhibited by MVP interacting with PERK (PRKR-like endoplasmic reticulum kinase) or through nuclear exportins illustrating the dynamic regulation of PTEN subcellular localization [139,140]. Furthermore, the different import systems used in different cell types also reveal the context dependence of these shuttling networks.

What are the molecular cues to cause PTEN to accumulate in the nucleus? Oxidative stress, including hypoxia, causes PTEN to accumulate in the nucleus [70,141,142]. Hypoxia causes nuclear PTEN to engage and protect p53 from degradation or inactivation in the nucleus [141]. This oxidative stress-induced nuclear PTEN accumulation is reversible and is controlled by PTEN’s monoubiquitylation at lysine 13 [70]. Oxidative stress also affects nuclear PTEN’s export rate, not its import rate [70,142]. Aside from oxidative stress, lower intracellular ATP levels or increased intracellular calcium concentrations, can also cause an increase in nuclear PTEN expression [142,143]. Both low intracellular ATP and high intracellular calcium concentrations are indicative of cells that are not healthy and may be approaching death [144,145].

### 3.3. Post-Translational Modifications Are Necessary for PTEN Nuclear Translocation and Stability

PTEN nuclear import is tightly regulated through a host of post-translational alterations including ubiquitination, phosphorylation, neddylation, SUMOylation, and oxidation [146]. These molecular additions provide the needed signaling inputs to trigger the trafficking of PTEN into the nucleus. Furthermore, these modifications also cause changes in PTEN’s stability both within the cytoplasm and the nucleus.

PTEN ubiquitination controls nuclear translocations. Simply tagging PTEN with a ubiquitin tag leads to PTEN nuclear localization [46]. Several ubiquitin ligases target PTEN for the addition of ubiquitin adducts. NEDD4-1 is an E3 ubiquitin ligase that can both mono- and poly-ubiquitinate PTEN at lysine 13 and 289; the mono-ubiquitination signal causes nuclear PTEN import and the poly-ubiquitination signal leads to cytoplasmic degradation [22,147]. Ubiquitination of PTEN at lysine 13 and 289 are important for IPO11-mediated transport of PTEN into the nucleus [132]. The E2 ubiquitin ligase UBE2E1 primes PTEN for cytoplasmic degradation, but IPO11 facilitates the transfer of the ubiquitin-loaded PTEN into the nucleus to protect PTEN from the NEDD4-1/NDFIP (NEDD4 family interacting protein) lysine ubiquitination system [132]. NDFIP1 is an E3 ubiquitin ligase adaptor protein and along with the small GTPase RAB5 regulates ubiquitinated PTEN nuclear trafficking [148,149]. Interestingly, RAB5 alone regulates the subcellular location of PTEN to endosomes aside from acting in concert with NDFIP1 to locate PTEN to the nucleus [148]. Other E3 ubiquitin ligases suggested to target PTEN to the nucleus include XIAP (X-linked inhibitor of apoptosis), SMURF1 (SMAD specific E3 ubiquitin protein ligase 1), and CHIP (C-terminus of the Hsc70-interacting protein) [150,151,152].

USP7 (ubiquitin specific peptidase 7) is a deubiquitinase (DUB) suggested as a major regulator in shifting nuclear PTEN to the cytoplasm. This is seen exquisitely in acute promyelocytic leukemia (APL) where PTEN is localized to the cytoplasm and the nucleus is devoid of PTEN due to USP7 [153]. USP7 is also found in the nucleus, but nuclear USP7 is indicative of an imbalance in the mono- and poly-ubiquitinated PTEN [120]. Another DUB, USP11, is critical in removing poly-ubiquitin from nuclear PTEN as opposed to single ubiquitin tags [154]. In contrast, the DUBs USP13 and OTUD3 (OUT deubiquitinase 3) enzymatic activity against ubiquitinated PTEN is exclusive to cytoplasmically located PTEN [154,155,156]. Developing a more complete molecular atlas of both ubiquitin ligases and deubiquitinases within their correct subcellular environments will be crucial in the development of a more complete compendium of PTEN function, especially considering that ubiquitinated PTEN may not be as catalytically active [157]. 

There is some debate as to whether NEDD4-1 is truly indispensable for PTEN to traverse the nuclear membrane [158]. It may be more plausible that NEDD4-1 is only regulating PTEN’s stability and not PTEN localization as the lysine 13 residue is critical for protein stability [23]. However, in a PHTS patient with ASD, the PTEN^I101T^ mutation found causes a decrease in both PTEN stability and PTEN nuclear abundance [31]. This may be due to nuclear PTEN’s half-life of only three hours, a half-life much shorter than bulk or cytoplasmic PTEN [71]. Nuclear PTEN stability is more tightly regulated by FBXO22 (F box protein 22) instead of NEDD4-1, WWP1, and WWP2 (WW domain containing E3 ubiquitin ligase 1 or 2) which regulate PTEN’s cytoplasmic degradation [71,79,159]. Rare PHTS mutations may prove invaluable in detangling phenotypes associated with either PTEN localization or stability.

Phosphorylation is another key regulator of PTEN stability and nuclear importation. The C-terminal domain of PTEN is phosphorylated at several residues by casein kinase II (CK2), GSK3β (glycogen synthase kinase 3β), RhoA kinase, and PI3K subunit p110δ [160,161,162,163,164,165]. Many of these phosphorylation sites are found in nuclear exclusion motifs that cause nuclear PTEN accumulation following plasma membrane disassociation [23,116,127]. There also may be a decrease in phosphatase activity following C-terminal phosphorylation [160]. PTEN’s phosphorylated carboxy terminus causes PTEN to be kept in a “closed” confirmation, whereas an “open” PTEN confirmation is critical for PTEN stabilization and plasma membrane binding or shuttling into the nucleus [23,166,167]. This “open” confirmation is necessary for ubiquitination to occur for the efficient transport of PTEN into the nucleus [23,46]. Furthermore, PTEN tyrosine 240 phosphorylation by FGFR2 (fibroblast growth factor receptor 2) also causes glioblastoma (GBM) cells to become more radioresistant and epidermal growth factor receptor tyrosine kinase inhibitor resistant [41,43]. These data show how critical phosphorylation is for maintaining PTEN stability and even treatment sensitivity.

Aside from ubiquitination and phosphorylation, SUMOylation also regulates PTEN subcellular localization. PTEN is SUMOylated at lysine 254 and SUMOylation promotes importation of PTEN into the nucleus in colon cancer or glioblastoma cell lines [168]. However, in prostate cancer models with hyperSUMOylation, there is an increase in cytoplasmic PTEN [169]. This suggests some context specificity in how SUMOylation regulates PTEN subcellular localization. Neddylation by NEDD8 or XIAP can also promote nuclear import [170]. The biochemical oxidation of cysteine 71 and 124 residues in PTEN also leads to both enzymatic inactivation, monoubiquitylation, and increased nuclear import in thyroid cancer cells expressing *SDHD* mutants *(succinate dehydrogenase subunit D*), another gene when mutated causes a PHTS-like syndrome [171,172]. 

**Figure 1:**
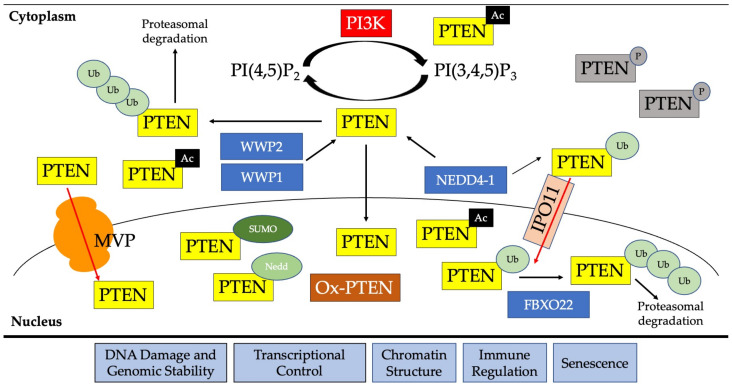
Schematic showing post-translational modifications important for PTEN nuclear import. PTEN canonically functions as a PI3K pathway negative regulator by dephosphorylating PI(3,4,5)P_3_. In the cytoplasm, mono- or poly-ubiquitination (Ub) by E3 ubiquitin ligases such as WWP2, WWP1, or NEDD4-1 can dictate nuclear import or proteasomal degradation, respectively. PTEN can be imported by the Major Vault Protein (MVP) or importins, such as IPO11. There is an abundance of SUMOylated (SUMO), neddylated (Nedd), and oxidized PTEN (Ox-PTEN) in the nucleus compared to the cytoplasm. In the cytoplasm, phosphorylated PTEN (P) is in more abundance. Acetylated (Ac) PTEN can be found in the nucleus, cytoplasm, or at the plasma membrane depending on the context that the acetylation is occurring. FBXO22 is the E3 ubiquitin ligase in the nucleus responsible for modulating nuclear proteasomal degradation.

Acetylation is another regulator of PTEN subcellular localization. PTEN is acetylated by PCAF (KAT2B, lysine acetyltransferase 2B) at K125 and K128; this acetylation may mediate nuclear PTEN import and increase AKT phosphorylation [173]. Hyperacetylation by deleting *SIRT1* (*Silent mating type information regulation 2 homolog 1*) in embryonic stem cells also led to decreased phosphatase function but mostly led PTEN to be excluded from the nucleus [174]. Intriguingly, K164 acetylation can enhance PTEN translocation to the membrane and increase its phosphatase activity [173]. PTEN K402 is also acetylated and a switch between acetylation and neddylation at this residue can alter plasma membrane or nuclear localization [170,175]. Acetylated K402 also impacts the affinity PTEN has for PDZ domains, increasing the interaction with MAGI2 [175]. Taken together, PTEN acetylation is a highly dynamic process that negatively regulates PTEN phosphatase function, but there are context dependencies that need to be considered when accounting for how acetylation affects subcellular localization. A summary of the different post-translational modifications that lead to PTEN nuclear import are found in Figure 1.

### 3.4. Nuclear and Cytoplasmic PTEN Partitioning In Vivo 

As for determining the cytoplasmic and nuclear PTEN functions in vivo, several germline mutant mice have been developed. In one mouse, deleting the PTEN C-terminus causes a decrease in murine lifespan due to increased tumor development, specifically within mammary tissue, thyroid, adrenal pheochromocytoma, and B cell lymphomas [176]. The PTEN C-terminus is a key hub for regulating nuclear PTEN import and maintaining chromosomal integrity [23,64,166,167,177]. Non-catalytic mutations in PTEN’s C2 domain, *Pten^F341V^*, also cause nuclear PTEN deficiency [50]. Homozygous *Pten^F341V/F341V^* die soon after birth due to an inability to intake milk [50]. Heterozygous *Pten^F341V/+^* mice, though, developed a similar but not overlapping tumor profile as germline *Pten* heterozygous mice with mammary tissue, thymus, adrenal gland, and stomach cancers observed, but an absence of uterine, thyroid, and prostate cancers observed in *Pten^+/−^* mice [34,50]. Nuclear PTEN is frequently phosphorylated at tyrosine 240, and this residue is critical for facilitating DNA repair [41]. Mice with germline *Pten^Y240F^* mutations are unable to efficiently repair DNA damage and are more sensitive to ionizing radiation [41]. 

Other nuclear PTEN-deficient germline models (*Pten^K13R,D384V^* or *Pten^K13R^*) exhibit microcephaly with decreased cerebral cortex, cerebellum, and hippocampus size, but brain mass is the only major change in organ size between wild-type and mutant mice [24,52]. In *Pten^K13R,D384V/-^* mice, cytoplasmic PTEN alone causes brain mass to approach wild-type PTEN expressing masses, but lymphomas are further exacerbated than what is seen in PTEN heterozygous and *Pten^K13R,D384V/K13R,D384V^* mice [178]. Following application of carcinogenic N-nitrosodiethylamine and hepatotoxin (CCl_4_), the combination of oxidative stress and no nuclear PTEN leads to the development of hepatocellular carcinoma further exacerbated p53 loss [70,179].

In another model of nuclear PTEN deficiency that has major implications in PHTS-related ASD, the *Pten^M3M4^* mouse has five different amino acid substitutions within exon 7 of *Pten*; exon 7 in *Pten* encodes the NLS-like domain rendering a more cytoplasmic dominant PTEN protein [38,180]. These mice develop macrocephaly, a common feature of PHTS patients, and increased cytosolic proteasomal activity [181,182]. Unlike catalytically dead PTEN mutant mice, the M3M4 mice can reach homozygosity [29,180,181,182,183,184,185,186]. Furthermore, *Pten^M3M4^* mice have behavioral and physiological profiles reminiscent of humans with ASD included dysregulated dopamine signaling, glial cell hyperproliferation, and leukodystrophy [180,183,185,186]. The homozygous *Pten^M3M4/M3M4^* mice also have a skewed B- and T-cell immune repertoires with deficits in central immune tolerance [29]. 

Conversely, nuclear predominant PTEN germline mutant mice (*Pten^Y68H^*) had similar immune repertoires and functions to wild-type PTEN-expressing mice [29]. Interestingly, the *Pten^Y68H^* mutant mouse also had behavioral phenotypes associated with human ASD, but different than what is seen with the *Pten^M3M4^* mice [180,185]. Mice expressing nuclear predominant PTEN had decreased sociability and interest in social novelty with increased repetitive behaviors and possible anxiety [185]. This contrasts with the cytoplasmic predominant PTEN-expressing mice who had increased social behaviors in male mice with both male and female mice exhibiting deficits in motor coordination and balance [180]. Taken together, this suite of mice with germline mutations causing either cytoplasmic or nuclear PTEN retention have indicated that there are PTEN compartment specific effects at the organismal level on tumorigenesis and neurodevelopment.

## 4. Nuclear PTEN Functions and Consequences of Their Loss in Cancer

PTEN loss facilitates many cancer phenotypes [1] (Table 2). Many of these phenotypes are associated with nuclear PTEN functions. These include regulating DNA damage while maintaining genome stability and integrity, suppressing oncogenic transcription, chromatin interactions, immune system dysregulation, and PTEN-induced cellular senescence. Undoubtedly, more cancer-related phenotypes associated with nuclear PTEN functions will be found.

### 4.1. DNA Damage Repair Regulation and Genomic Integrity Maintenance by Nuclear PTEN

Nuclear PTEN facilitates the resolution of damaged DNA in order to maintain chromosomal integrity. When PTEN expression or activity is lost in several different tumor and tissue types, DNA damage, specifically double-stranded breaks, is increased [50,109,131,177,208]. DNA damage resolution is controlled by post-translational modifications on the PTEN protein, namely methylation, phosphorylation, and SUMOylation. PTEN is demethylated at K349 by NSD2 (nuclear receptor binding SET domain protein 2); this dimethyl mark is recognized by the tudor domain on 53BP1 (tumor protein 53 binding protein 1) and recruited to areas of DNA damage within the cell to facilitate repair [187]. PTEN also acts in concert with the p53 pathway (via p53 or p73) in order to resolve damaged DNA [142,192,193]. SUMOylation-deficient PTEN also cannot resolve DNA damage; increased 53BP1, BRCA1, and λH2AX foci and reduced chromatin recruited RAD51 in ionizing radiation-treated cells are indicative of unresolved, damaged DNA [168]. Phosphorylation at multiple residues in PTEN’s C-terminal tail is implicated for DNA double-stranded break resolution by recruiting RAD51 or RAD52 to sites of DNA damage [188,189]. Indeed, by truncating PTEN’s entire C-terminus, there is an increase in DNA double stranded breaks [177]. The ATM (ataxia-telangiectasia mutated) kinase is a key regulator of C-terminal phosphorylation, thusly engaging these DNA repair pathways [50,168,187]. Nuclear-localized PTEN frequently facilitates homologous recombination (HR) in order to repair DNA double stranded breaks, as evidenced by PTEN-RAD51 interactions [41,168,177,188,190,191]. Non-homologous end-joining (NHEJ) can also be utilized by nuclear PTEN to repair DNA but is frequently repressed by blocking Ku70 recruitment to areas of DNA damage [168,187,190]. Aside from not resolving deleterious DNA damage, PTEN loss also increases single strand annealing, another DNA damage repair pathway with a high error rate, potentially compounding the unresolved DNA damage seen when PTEN activity is lost [191].

PTEN-deficient cells also see catastrophic chromosomal damage. Nuclear PTEN decreases the number of micronuclei in cells [194]. Furthermore, the loss of the PTEN interaction with kinetochore component CENP-C (centromere protein C) leads to centromere breaking [177]. Genomic stability is also lost in PTEN null cells due to unrestrained replication fork progression, as seen after aphidicolin treatment in prostate cancer cells [195]. Indeed, PTEN interacts with replication fork component MCM2 (minichromosome maintenance complex component 2) to restrict replication fork progression [196]. PTEN thusly helps facilitate DNA damage responses during DNA synthesis [209]. The PTEN C-terminal tail also appears to be critical in maintaining chromosomal integrity [176].

Taken together, PTEN loss within the nuclear compartment has detrimental effects on the cell’s ability to repair DNA damage. However, this does create potential therapeutic vulnerabilities. PTEN is a synthetic lethal partner with PARP (poly-ADP-ribose polymerase) inhibitors [109,191,210]. This could also coincide with increased reliance on ATM kinase inhibitors given the major role of this kinase on maintaining these DNA damage repair pathways [50,168,187]. These pharmacological interventions could be beneficial for stratifying patients with nuclear PTEN-deficient cancers. 

### 4.2. PTEN and Transcriptional Control 

Unlocking phenotypic plasticity is an emerging cancer hallmark [1]. Phenotypic plasticity is centered around the adaptability of transcriptional programs to evade the terminal differentiation programs needed to be usurped in carcinogenesis [1,211]. PTEN is a major regulator of early embryogenic, mammary, neuronal, and smooth muscle tissue differentiation and prevents a lineage switch between basal and luminal cells in the prostate [3,102,103,104,197,212]. Increasing evidence points toward PTEN as a major regulator of transcription and differentiation whose loss causes aberrant oncogenic transcriptional programs to be implemented. 

One key relationship between PTEN loss and tumor promoting transcriptional programs seen in rare cancers is the PTEN–PAX7 (Paired box 7) axis observed in both glioblastoma and rhabdomyosarcoma. In both instances, PTEN loss unlocks a transcriptional network centered on the skeletal muscle satellite cell marker PAX7 [68,198]. When *PTEN* is lost in human neural stem cells, there is an increase in phosphorylated CREB (cyclic AMP response element binding protein) leading to increased *PAX7* transcription and ultimately a more glioblastoma-like cell state [68]. In rhabdomyosarcoma, *Pten* loss caused a less differentiated, more aggressive tumor dependent on PAX7 for not only the malignant phenotype but for rhabdomyosarcoma identity itself [198]. This axis illustrates how PTEN loss activates tumorigenic transcriptional programs.

PTEN interacts with several members of the transcriptional machinery, including AFF4 (ALF transcription elongation factor 4), cyclin T1, XPB (ERCC3, ERCC excision repair 3), CDK7 (cyclin-dependent kinase 7), and RNA polymerase II, highlighting how important nuclear PTEN is in regulating transcriptional processes [199]. PTEN binds and dephosphorylates serine 5 on the C-terminal tail of RNA polymerase II, and loss of PTEN leads to global increases in RNA polymerase II occupancy at promoters [200]. In mice with PTEN predominantly expressed in the nucleus, the brain cortex shows a shift toward gene over expression instead of under expressed genes [185]. This may indicate that nuclear PTEN may negatively regulate transcriptional repressors. By overexpressing PTEN with the “Super PTEN” mouse, transcriptional levels can be lowered as evidenced by decreased MYC [213]. MYC rapidly increases cellular RNA and amplifies gene expression bursts throughout the genome [214,215]. Together, this global transcriptional dysregulation caused by PTEN deficiency can be a major oncogenic driver. Furthermore, increased transcription can also cause DNA damage and genomic instability which will be exacerbated in cancers without nuclear PTEN present [216,217].

Splicing is also regulated by nuclear PTEN. Alternative splicing can be used by cancer cells to become more transcriptionally plastic [218]. In the *Pten^M3M4^*-cytoplasmic dominant mouse, alternative splicing is disrupted and possibly may cause the deficits in central immune tolerance in these mice [29,201]. The aberrant splicing is due to disruptions in PTEN interacting with the splicing machinery, specifically U2AF2 [202]. Decreased PTEN expression caused the inclusion of an aberrant exon in GOLGA2 which caused tumorigenic changes in prostate cancer cells [202]. 

### 4.3. Chromatin Interactions with Nuclear PTEN 

Despite not having a DNA-binding domain, PTEN interacts with chromatin in multifaceted, dynamic ways [199,219]. Ionizing radiation or other genotoxic stresses cause a strengthened PTEN-chromatin interaction; this protein-DNA complex is necessary to recruit the DNA damage repair machinery, such as RAD51 or RAD52, to areas where double-stranded DNA breaks have occurred [41,203]. Furthermore, removing PTEN from chromatin is critical for daughter cells to enter the G1 phase of the next cell cycle [189]. PTEN’s C-terminus is critical in coordinating both chromatin interactions [189,203,204].

PTEN also affects chromatin structure. PTEN loss causes a reduction in H3K9me3 indicative of heterochromatin decondensation in a human osteosarcoma cell line [205]. Much like in facilitating DNA damage or movement through the cell cycle, the C-terminal domain of PTEN is also critical in heterochromatin maintenance via interacting with heterochromatin protein 1α (HP1α) [204,205]. Histone 1 (H1) interacts with the PTEN C2-domain in a phosphatase independent manner, and the loss of PTEN expression causes an increase in H1 motility and lost chromatin organization [204]. The lost chromatin organization is manifested by increased H4K16 acetylation, a marker of decondensed chromatin; this increased H4K16 acetylation deposition causes further impairment of the H1-PTEN interaction [204]. PTEN also controls oncogenic Histone 3.3 (H3.3) deposition by forming a repressive complex with H3.3 and DAXX (death domain associated protein) in GBMs [206]. This suggests that a major role for nuclear PTEN role in suppressing tumorigenesis occurs through regulating chromatin stability and dynamics. This regulation centered on PTEN may provide therapeutic benefits as chromatin interactors, BRG1 (SMARCA4, SWI/SNF related, matrix associated, actin dependent regulator of chromatin, subfamily A, member 4) and CHD1 (chromodomain helicase DNA binding protein 1), act synthetic lethally with PTEN [220,221]. The CHD1-PTEN synthetic lethal relationship may even be exploited further using immune checkpoint inhibition given CHD1’s role in maintaining an elevated level of myeloid-derived suppressor cells in *Pten*-deficient prostate cancers [222].

### 4.4. Nuclear PTEN, the Immune System, and Cancer

DNA damage is frequently associated with altered immune signaling [223]. PHTS patients exhibit immune dysfunction including peripheral blood lymphopenia, lymphoid hyperplasia, and autoimmunity [224]. Indeed, PTEN loss of expression, both throughout the cell and the nuclear compartment is associated with altered immune signaling and immune cell profiles [29,225,226]. The increase in unresolved DNA damage and genomic instability in nuclear PTEN-deficient cells may contribute some to some of this dysregulation; indeed, nuclear PTEN loss may lead to both immunosuppressive and immunoresponsive tumor microenvironments [207]. Understanding more about how nuclear PTEN augments tumor immunogenicity will be a critical element of understanding PTEN’s full tumor-suppressive capacity. 

### 4.5. Cellular Senescence and Nuclear PTEN

Acute PTEN loss causes cells to enter senescence in a p53-dependent manner [227]. Ionizing radiation can also cause mutant PTEN cells to become senescent as opposed to undergoing apoptosis in wild-type PTEN expressing cells [228]. Cellular senescence is triggered due to dysregulation of the nuclear PTEN-CDH1-APC complex (cdc20 homolog 1-anaphase promoting complex) [67]. This senescence phenotype appears distinct from oncogene-induced senescence as it occurs in the absence of proliferation and DNA damage [229]. However, these PTEN-deficient senescing cells can cause an immunosuppressive microenvironment to form due to the increased senescent-associated secretory phenotype caused by increased JAK/STAT (Janus kinases/signal transducer and activator of transcription) signaling [230].

## 5. Concluding Remarks

PTEN is normally located in both the cytoplasm and the nucleus, and there is mounting evidence that disrupted PTEN subcellular localization is tumorigenic. Furthermore, disrupted PTEN subcellular localization cannot be accounted for using next-generation sequencing technology. Therefore, understanding how dysregulated PTEN compartmentalization contributes to oncogenesis is necessary to comprehend the many facets of PTEN function. There is still much to learn in this regard. There is an entire PI3K pathway ecosystem found in the nucleus independent of its cytoplasmic counterpart [192,231]. The distinctive roles for PTEN in the cytoplasmic and nuclear PI3K pathways remains poorly understood. There is also an increasing repertoire of PTEN isoforms originating from alternative start codons that show differential subcellular localization [72,232,233,234,235,236]. Examining how these different isoforms interact with the wild-type PTEN protein in more cellular and disease contexts is still needed. These include rare cancers, where the paucity of model systems has hindered basic, translational, and clinical advances. Therefore, determining how PTEN subcellular localization affects these rare diseases is even more critical.

## Figures and Tables

**Table 1:** PTEN mutations and pathophysiological effects associated with each mutation that are discussed in this review.

PTEN Mutation	Physiological Effect	In COSMIC or SFARI? ^1^	Germline or Somatic? ^2^
K13E	Nuclear excluded [22]; less stable than wild-type PTEN [23]	COSMIC	Somatic
K13R	Nuclear excluded, analogous to K13E [24]	Neither	N/A
T26I	Nuclear excluded [25]	Both	Germline and Somatic
G44D	Nuclear predominant [26]; mostly lipid phosphatase dead [27]	COSMIC, not in SFARI but reported elsewhere [26,27,28]	Germline and Somatic
Y68H	Nuclear predominant [29]	COSMIC, not in SFARI but reported elsewhere [28]	Germline and Somatic
P95R	Nuclear excluded [25]	Neither, but reported by [25]	Germline (PHTS, macrocephaly) [25]
I101T	Decreased stability, lipid phosphatase activity, and nuclear localization [30,31]	Both	Germline and Somatic
H123Q	Nuclear predominant [26]; partial loss of lipid phosphatase activity [27]	COSMIC, not in SFARI but reported elsewhere [26,32]	Germline and Somatic
C124S	Catalytically dead (both lipid and protein) [33]; possibly less stable than wild-type PTEN [34,35]	COSMIC	Somatic
G129E	Lipid phosphatase dead [35]	COSMIC	Germline and Somatic
Y138L	Protein phosphatase dead [36]	Neither	Neither; Other Y138 mutations observed [37]
E157G	Nuclear predominant [26]	Neither	Germline and Somatic (ASD) [26,28]
Y177N	Nuclear excluded and decreased stability [25]	Neither, but reported by [25]	Germline (PHTS, macrocephaly) [25]
R234Q	One of the M3M4 ^3^ mutations [38,39]; Plasticity in tumor lineage [40]	COSMIC	Germline and Somatic
Y240F	Unable to efficiently repair DNA damage, less nuclear PTEN [41]; decreased lipid phosphatase activity [42]: Epidermal growth factor receptor inhibitor resistance [43]	Neither	N/A
F241S	Nuclear excluded, but decreased protein stability [30,44]	COSMIC, not in SFARI but reported elsewhere [44]	Germline and Somatic
D252G	Nuclear excluded, but decreased protein stability [30,44]	COSMIC, not in SFARI (D252V is seen in SFARI); reported elsewhere [28]	Germline and Somatic
Q261E	Nuclear excluded [25]	COSMIC, not in SFARI but reported elsewhere [25]	Germline (PHTS, macrocephaly and lipomas), unknown Somatic (as noted in COSMIC) [25]
W274L	Nuclear excluded [44]	COSMIC, not in SFARI (W274G is noted in SFARI); reported elsewhere [27,28]	Germline; unknown Somatic (as noted in COSMIC)
N276S	Nuclear excluded, but decreased protein stability [30,44]	COSMIC, not in SFARI but reported elsewhere [30,44,45]	Germline and Somatic
T277A	Nuclear and cell membrane excluded; decreased protein stability [25,46]	COSMIC, not in SFARI (T277I is noted in SFARI), reported elsewhere [25]	Germline and Somatic
K289E	Nuclear excluded [22]	COSMIC	Germline and Somatic
D310G	Nuclear excluded [25]	COSMIC (also reported in [47]); not in SFARI but reported elsewhere [25]	Germline and Somatic
D326N	Nuclear predominant [31]; perhaps decreased stability [28,48]	Neither, but reported by [31,49]	Germline and Somatic
F341V	Nuclear excluded, decreased stability, decreased phosphatase activity [50,51]	COSMIC	Germline (as noted in [50]) and Somatic
D384V	Nuclear excluded [52]	Neither	N/A
M3M4 ^3^	Collection of mutations (see note 3) in murine model of PTEN nuclear exclusion [38]	N/A	N/A

^1^ Data obtained from COSMIC or SFARI (Simons Foundation Autism Research Initiative) databases in January 2023. ^2^ Data obtained from COSMIC or SFARI databases in January 2023. ^3^ M3M4 is a designation for a series of missense mutations (R233Q, R234Q, K266N, and K267Q) in exon 7 of murine *Pten* [38].

**Table 2:** Nuclear PTEN Functions in Cancer.

Nuclear PTEN Functions	Summary of Nuclear PTEN Functions	Reference
DNA damage repair and genomic integrity maintenance	Repair of double-stranded DNA breaks; promotes homologous recombination (HR) and either promotes or restrains non-homologous end-joining (NHEJ) possibly by impeding Ku70 binding to DNA breaks; PTEN Y240 promotes DNA damage repair by facilitating RAD51 filament formation and stabilization; SUMOylated PTEN required for repair; ATM phosphorylates the C-terminal tail of PTEN to facilitate DNA damage repair; NSD2-mediated dimethylation at K349 and PTEN protein phosphatase domain activity are required for DNA damage repair	[41,50,109,131,168,177,187,188,189,190,191]
	Works in concert with p53 or p73 to regulate DNA damage repair response	[142,192,193]
	Increased single strand annealing when PTEN lost	[191]
	Protects cells from catastrophic genomic instability by binding to centromeric CENP-C	[176,177,194]
	Restricts replication fork progression by stabilizing heterochromatin or dephosphorylation MCM2	[195,196]
Transcriptional control	Regulates tissue differentiation, i.e., neuronal, mammary, smooth muscle	[102,103,104,197]
	Represses aberrant transcriptional programs, by dephosphorylating CREB	[68,198]
	Interactions with the transcriptional machinery, i.e., RNA polymerase II	[199,200]
	Regulation of alternative splicing through binding to U2AF2 and other spliceosome proteins	[29,201,202]
Chromatin structure	Interactions necessary to facilitate DNA damage repair; PTEN Y240 binds to chromatin via Ki67 to regulate DNA damage repair; binds with SUMOylated Rad52 on chromatin	[41,203]
	Heterochromatin structure stabilization, such as binding to HP1α; dependent on PTEN C-terminus interacting with Histone H1	[195,204,205]
	PLK1-mediated phosphorylation regulates chromatin PTEN, removed by Cdh1	[189]
	Oncogenic histone modifications (represses oncogenic DAXX-H3.3. interaction) and deposition (increased H4K16ac opens chromatin when PTEN-Histone H1 interaction is disrupted)	[204,206]
Immune regulation	Altered immune profiles (i.e., central immune tolerance and both pro- and anti-inflammatory responses)	[29,207]
Senescence	Promoting pro-senescent E3 ubiquitin ligase activity	[67]

## Data Availability

Not applicable.
